# The Promise of Explainable AI in Digital Health for Precision Medicine: A Systematic Review

**DOI:** 10.3390/jpm14030277

**Published:** 2024-03-01

**Authors:** Ben Allen

**Affiliations:** Department of Psychology, University of Kansas, Lawrence, KS 66045, USA; benallen@ku.edu

**Keywords:** digital health, explainable artificial intelligence, precision medicine, machine learning

## Abstract

This review synthesizes the literature on explaining machine-learning models for digital health data in precision medicine. As healthcare increasingly tailors treatments to individual characteristics, the integration of artificial intelligence with digital health data becomes crucial. Leveraging a topic-modeling approach, this paper distills the key themes of 27 journal articles. We included peer-reviewed journal articles written in English, with no time constraints on the search. A Google Scholar search, conducted up to 19 September 2023, yielded 27 journal articles. Through a topic-modeling approach, the identified topics encompassed optimizing patient healthcare through data-driven medicine, predictive modeling with data and algorithms, predicting diseases with deep learning of biomedical data, and machine learning in medicine. This review delves into specific applications of explainable artificial intelligence, emphasizing its role in fostering transparency, accountability, and trust within the healthcare domain. Our review highlights the necessity for further development and validation of explanation methods to advance precision healthcare delivery.

## 1. Introduction

Precision medicine is a way of personalizing treatments and interventions to the patient’s characteristics, such as genetics, environment, and lifestyle [1]. This personalized medicine is a shift in healthcare to the use of information unique to the patient as a guide for diagnosis and prognosis [2]. Precision medicine has the allure of increasing the reach of medical treatment beyond the one-size-fits-all approach, especially when leveraging advanced bioinformatic strategies to interpret and apply clinical data and provide patients with customized medical care [3]. Precision medicine has the potential to make healthcare more efficient and effective [4].

A major catalyst toward the success of personalized medicine is the integration of different forms of digital healthcare data with artificial intelligence to make more accurate interpretations of diagnostic information, reduce medical errors, and improve health system workflow and promote health [5]. A noteworthy example comes from a study of an artificial-intelligence system trained to suggest different chemotherapy treatments based on the predicted treatment response given the patient gene-expression data [6]. The prediction models showed accuracy near 80% and might eventually help cancer patients avoid failing therapies. There are similar studies of artificial-intelligence systems trained to suggest different antidepressant treatments based on digital health records [7]. Overall, these studies suggest that clinical support systems could help personalize healthcare delivery when given the right reservoir of digital health data [8].

Digital health technologies are a rich reservoir of big health data for personalizing medicine. For example, wearable biosensors can measure valuable health-related physiological data for patient monitoring and management [9]. Telemedicine also has the potential to make healthcare more cost-effective while meeting the increasing demand for and insufficient supply of healthcare providers [10]. Overall, artificial-intelligence applications can leverage digital health data to implement personalized treatment strategies [1,5].

One of the key challenges in implementing precision medicine is integrating diverse and comprehensive data sources that encompass genetic, environmental, and lifestyle factors to ensure healthcare systems improve patient outcomes and effectively manage diseases [1,4]. The integration of complex datasets can be an overwhelming task for even a team of humans, but is relatively trivial for artificial-intelligence systems. Evidence of such integration is demonstrated in machine-learning models trained to make breast cancer diagnoses using digital health records along with analysis of mammography images [11]. Notably the prediction model showed a specificity of 77% and sensitivity of 87%, suggesting potential for reducing false-negatives. As artificial-intelligence systems become more prevalent in healthcare, such systems will be able to leverage genetic, environmental, and lifestyle data to help advance a personalized medicine approach [12].

Integrating important health data requires responsible handling and safeguards to prevent misuse of protected health information [13]. More recently, integrating and interpreting complex health data for personalized medicine is becoming the job of artificial intelligence [5]. Yet, developing countries may have limited access to these artificial-intelligence applications, highlighting the importance of open-source code [14]. Moreover, there can be misuse of health information embedded in prediction models used by artificial-intelligence systems to extract patterns from data to inform medical decisions [15]. As artificial-intelligence applications become more prevalent in healthcare, there is a great need to ensure ethical issues are considered along the way [16].

Integrating artificial intelligence into healthcare offers several potential benefits, including more accurate diagnostic/prognostic tools, more efficient personalization of treatment strategies using big data, and overall better optimization of healthcare workflows [5,17]. The sheer volume of patient health data available also makes integration via artificial intelligence into healthcare a necessity. Artificial-intelligence systems can quickly extract meaningful patterns and insights from multiple data sources, enabling better-informed decisions about how to personalize healthcare [15].

But the desire for accurate artificial-intelligence systems must be balanced with the goal of transparency and interpretability to build trust among healthcare practitioners and patients and ensure the responsible integration of insights into clinical decision-making [18]. It is important to foster a collaborative approach between human expertise and machine intelligence by understanding an artificial-intelligence-system’s rationale when making medical decisions [5]. The rising field of explainable artificial intelligence centers around the ability to comprehend and interpret artificial-intelligence systems [19]. Explainable artificial intelligence promotes trust through transparency and accountability in artificial-intelligence applications for healthcare [17]. For precision medicine, healthcare practitioners are more likely to trust in the outcome of complex algorithms they can understand, giving explainable methods a position to ensure transparent models for personalized treatment strategies [19,20].

This review is a critical evaluation of the literature on how explainable artificial intelligence can facilitate the pursuit of precision medicine using digital health data. A secondary objective was to offer key strategies and knowledge gaps in addressing the challenges in interpretability and transparency of artificial-intelligence systems for precision medicine using digital health data. The primary inquiry addressed in this review was discerning the core themes and the status of research at the confluence of digital health, precision medicine, and explainable artificial-intelligence methodologies. This systematic review serves to pinpoint the benefits and challenges of applying explainable artificial-intelligence methods with digital health data for precision medicine.

This paper consolidates recent literature and offers a comprehensive synthesis of how to apply explainable artificial-intelligence methods to the utilization of digital health data in precision medicine. Machine learning is an effective approach to identifying treatment targets and accurately predicting treatment outcomes [21]. For example, there is evidence for using an artificial-intelligence-based system to select patients for intervention using the electrocardiograph signal to predict atrial fibrillation [22]. Employing a topic-modeling approach, this study extracted key themes and emerging trends from the literature on using explainable artificial intelligence and digital health data for precision medicine. Topic modeling is an unsupervised learning method for uncovering prevalent themes within a body of text [23,24]. Therefore, this paper provides a compilation for precision medicine of explainable artificial intelligence approaches to digital health.

## 2. Materials and Methods

### 2.1. Topic-Modeling-Procedure Overview

Insights derived from a topic-modeling analysis of the relevant literature directed this review. Specifically, the latent Dirichlet allocation (LDA) algorithm helped uncover prevalent themes in a final corpus of journal articles by analyzing the probability patterns of words and word pairs across the documents. The methods outlined in the subsequent sections followed the PRISMA 2020 checklist and were pre-registered on the Open Science Foundation (https://osf.io/tpxh6, registered on 19 September 2023) [25]. The Supplementary Materials include the checklist and code for the data analysis is available at https://zenodo.org/records/10398384, accessed on 19 September 2023.

### 2.2. Journal Article Search Strategy

**Figure 1:**
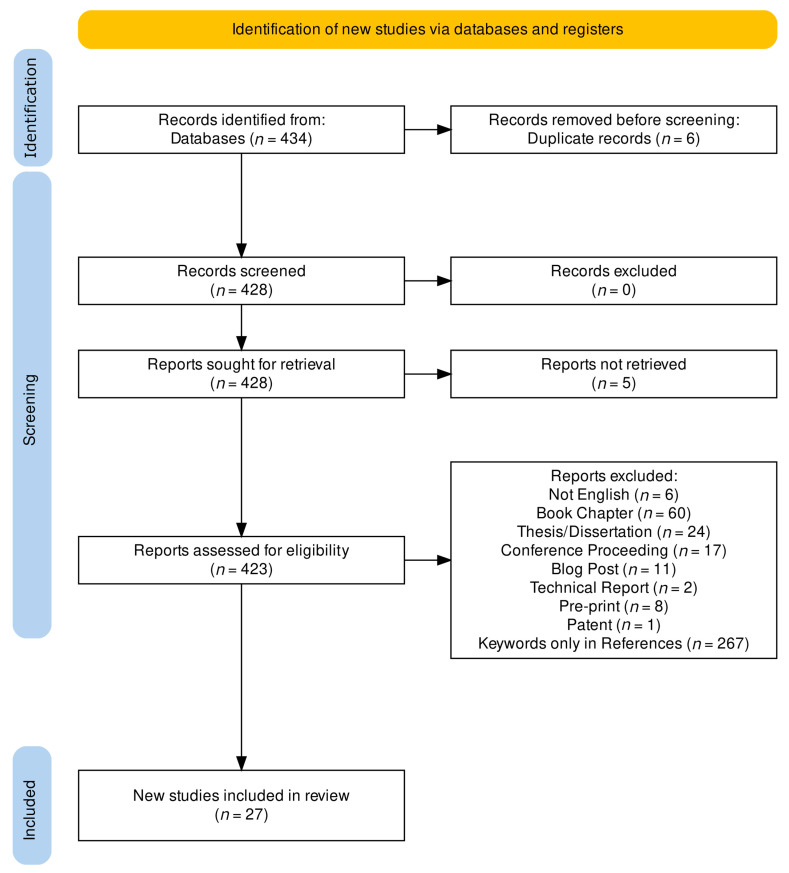
PRISMA 2020 flowchart. https://estech.shinyapps.io/prisma_flowdiagram/ (accessed on 1 January 2024.

A google scholar search (accessed on 19 September 2023) identified 434 journal articles relevant to this review. The search terms included: (“precision medicine” AND “digital health” AND “interpretable machine learning” OR “explainable artificial intelligence”). Inclusion criteria were that the article be written in English and was a peer-reviewed journal article, and full text was available, and included the search terms in the body of the text. The search was not restricted by date, though the earliest article matching our search terms was published in 2018. Citations and full-text articles were imported to the Zotero reference management software (https://www.zotero.org/). Zotero automatically classifies articles by type (i.e., journal article, pre-print, thesis, etc.). Each article’s classification was verified by the author. To screen for keywords in the text body, the reference sections were removed from each article, and spelling and grammar were checked through Google Docs. From each journal article, we extracted bigrams (consecutive word pairs). Articles only containing search terms in the reference section were excluded. Figure 1 shows the PRISMA 2020 flowchart which illustrates how the final set of articles was determined [26]. Table 1 shows the resulting 27 articles that directly connected explainable artificial intelligence to digital health and precision medicine.

### 2.3. Topic Modeling R

All text analysis and pre-processing occurred through the R programming language (version 4.3.1, 16 June 2023). We used the full text of each journal article, with the reference sections deleted; articles were segmented into paragraphs (*n* = 1733). The paragraphs were pre-processed by deleting punctuation, numbers, stop words, and symbols using the *tm* R package (version 0.7-8). Finally, we lemmatized each word and tokenized the text into unigrams, bigrams, and trigrams. This process helped combine counts of similar words with slightly different spellings. We removed 1 paragraph with fewer than 5 terms and removed all terms that occurred in only 1 paragraph (*n* = 241,027), resulting in 1732 paragraphs and 262 unique terms.

Using the *ldatuning* R package (version 1.0.2), we calculated coherence metrics for topic models of various sizes to estimate the optimal number of topics inherent to the collection of paragraphs. Next, we randomly split paragraphs into ten subsets, computed coherence metrics for topic models ranging from 2 to 20 topics, and repeated the process ten times to prevent bias. The median coherence scores across iterations suggested that a 5-topic model was optimal based on coherence. Subsequently, we employed the Gibbs algorithm to estimate a 5-topic latent Dirichlet allocation model for the entire corpus.

## 3. Results

Using a latent Dirichlet allocation model, we built a five-topic model based on the corpus of 27 journal articles that matched search terms. As topic modeling is unsupervised machine learning, one of the identified topics did not directly relate to the keywords. It identified a segment of paragraphs that described methods used to conduct literature reviews. That topic is omitted from the results below. The remaining four topics are discussed below based on an evaluation of each topic’s most probable n-grams, 100 most probable paragraphs, and their parent paper’s findings on precision medicine, digital health, and explainable artificial intelligence. The last two topics are merged under one heading because they are both related to deep learning and explainable artificial-intelligence research.

### 3.1. AI Explainability Addresses Ethical Challenges in Healthcare

Artificial intelligence (AI) is integral to offering solutions to various challenges in healthcare, including the standardization of digital health applications and ethical concerns related to patient data use [27,28]. Precision medicine, a common application of AI in digital health, involves tailoring healthcare interventions to subgroups of patients by using prediction models trained on patient characteristics and contextual factors [29,30,31]. However, the reliance on AI in healthcare raises issues regarding transparency and accountability with black-box AI systems whose decision-making processes are opaque [32,33]. Explainable artificial intelligence emerges as a solution to enhance transparency, ensuring that AI-driven decisions are comprehensible to healthcare providers and patients alike [29,34].

Explainable artificial intelligence provides explanations that increase the trustworthiness in the diagnoses and treatments suggested by machine-learning models [32,34,35,36]. While accuracy is necessary in AI systems, healthcare is a critical domain and requires transparent AI systems that offer reliable explanations [28,37]. When combined with rigorous internal and external validation, explainable artificial intelligence can improve model troubleshooting and system auditing, aligning the AI system with potential regulatory requirements, such as those outlined in the regulations on automated artificial-intelligence systems put forth by the European Union [37,38,39].

AI is well-suited to help precision medicine by computing mathematical mappings of the connections between patient characteristics and personalized treatment strategies [40,41,42,43]. However, challenges persist in the validation of machine-learning models for clinical applications [29,44]. Public and private collaborative efforts involving clinicians, computer scientists, and statisticians are essential to effectively map a machine-learning model onto an explanation that can be understood in the service of precision medicine [40,45].

There are going to be ever-present ethical and social concerns, including issues of accountability, data privacy, and bias [32,46]. Explainable artificial intelligence offers a pathway to addressing these concerns by providing transparent explanations for AI-driven decisions, fostering trust and acceptance among stakeholders [47,48]. Differences between machine-learning models trained on data from practical application vs. proxies make it challenging to have a unitary assessment of interpretability or explainability [49]. As AI continues to grow, there is an ongoing ethical need for the development of explainable artificial-intelligence methods in healthcare [17,50].

### 3.2. Integrating Explainable AI in Healthcare for Trustworthy Precision Medicine

Integrating explainable artificial intelligence with digital health data is gaining momentum in precision medicine, addressing the need for transparent and understandable models essential for clinical applicability [19,51,52]. As machine-learning models become more complex, interpretability is crucial in clinical contexts such as microbiome research [51,53]. Explainable artificial-intelligence applications can help predict an Alzheimer’s disease diagnosis in a pool of patients with mild impairments, showcasing how interpretable machine-learning algorithms can help explain complex patterns that inform individual patient predictions [54,55]. Such models offer patient-level interpretations, aiding clinicians and patients in understanding the patterns of features that predict conversion to dementia, thus enhancing trust in using explainable artificial intelligence as an aid to medical decisions [54,56].

Methods of extracting explanations from complex models can aid in the discovery of new personalized approaches to therapy and new biomarkers [57]. For example, Bayesian networks may serve as a framework for visualizing interactions between biological entities (taxa, genes, metabolites) within a specific environment (human gut) over time [51]. A model agnostic approach to explainability is offered by Shapley additive explanations, which enhance understanding at both global and local levels, improve predictive accuracy, and facilitate informed medical decisions [56,58]. Shapley values can enable visual explanations of how a model makes patient-level predictions and also the impact of changes in training data on model explanations [59]. Yet, a key barrier to advances of AI in healthcare in integrating data across platforms and institutions for precision medicine is the lack of clear governance frameworks for the privacy and security of data [60].

Development of AI systems for disease identification, such as in COVID-19 diagnosis, are underway, highlighting the importance of visual explanations in optimizing diagnostic accuracy [58,61]. For example, a recent study used explainable artificial-intelligence methods to create a multi-modal (visual, text) explanation as an aid in understanding and trusting a melanoma diagnosis [62]. More broadly, explainable artificial intelligence has potential to aid in communicating transparent decision support for healthcare systems that helps healthcare professionals make informed and reliable decisions [58,63]. Moreover, many legal and technological challenges associated with diagnostic models of electronic health records are solved by sharing prediction models and Bayesian networks of comorbidities on health outcomes, rather than the protected health information itself [64]. Overall, explainable artificial-intelligence methods are important for building trustworthiness for AI healthcare systems, supporting advancements in precision medicine and clinical decision-making [49,58,65].

### 3.3. Advancing Precision Medicine through Deep Learning and Explainable Artificial Intelligence

The great potential of deep learning is as a transformative force in the analysis of health information for precision medicine because of its ability to find patterns in unstructured data, such as images from medical scans, that are important for diagnosis and treatment decisions [66,67]. This ability has advanced the field, enabling the differentiation of medical conditions with high accuracy, as shown in studies comparing benign nevus and melanoma through skin-lesion images [66,68]. Explainable artificial-intelligence approaches to understanding clinical systems using deep learning offer explanatory metrics that can be used in validation studies, and help address ethical considerations and regulatory compliance [56,66].

Deep-learning models combined with explainable artificial intelligence have potential for broad applications in precision medicine, from enhancing disease diagnosis to facilitating drug discovery [69,70,71]. Deep-learning models offer more exact and efficient diagnosis for diseases requiring analysis of medical images (i.e., cancer, dementia), compared with human experts [72]. Explainable artificial-intelligence approaches to deep-learning models of medical images often include some form of visual explanation highlighting the image segments the model used to make the diagnosis [73,74]. Deep learning can also reduce drug discovery costs by efficiently screening for potential candidates, reducing time compared with traditional methods [29].

Deep-learning models for detecting, segmenting, and classifying biomedical images have accuracy that sometimes meets or exceeds human experts [75,76]. Multimodal data-fusion techniques that combine medical imaging data with other data sources show further improved diagnostic accuracy [77]. Explainable artificial intelligence makes AI algorithms more transparent and controllable, building trust among medical professionals in AI-assisted decisions [78]. Overall, explainable artificial intelligence integration into the healthcare systems can build trust and reliance in deep-learning approaches to diagnosis and drug discovery [56,69,79].

## 4. Discussion

This review paper gives an overview of key themes in research into digital health using explainable artificial intelligence for precision medicine. We used a topic-modeling approach to extract common themes across 27 full-text journal articles matching search criteria (“precision medicine” AND “digital health” AND “interpretable machine learning” OR “explainable artificial intelligence”). Thus, this review offers a glimpse at the current landscape in explainable artificial-intelligence-driven precision medicine using digital health. Through applying a latent Dirichlet allocation model, the topic model highlights core thematic areas that underscore an emerging focus on explainable artificial intelligence as a key to addressing ethical challenges [27,29,34,58,66,80,81,82]. These challenges include transparency, trust, and interdisciplinary collaboration in advancing healthcare innovations. Explainable artificial intelligence has many qualities that bridge the gap between complex AI algorithms, such as deep learning, and their practical applications in healthcare, enhancing the acceptability and effectiveness of AI interventions in clinical settings [19,51,52]. By facilitating a better understanding of AI-driven predictions, explainable artificial intelligence enables healthcare professionals to make informed decisions, thus fostering a collaborative environment where AI serves as a supportive tool for opaque decision-making [40,45]. The high-stakes clinical context makes it crucial to integrate explainable artificial intelligence into healthcare systems for advancing personalized treatment strategies that are grounded in an understanding of AI-generated insights.

The alliance between deep learning and explainable artificial intelligence is also critical for advancing precision medicine [66,67]. Deep learning can analyze medical imaging data with electronic health records. Coupled with the explanatory power of explainable artificial intelligence, it offers unprecedented opportunities for diagnosing and treating diseases with greater precision [83]. Explainable artificial intelligence increases accessibility and trust by medical professionals by enhancing the credibility and applicability of deep-learning models in healthcare [56,69,79]. This synergy between deep learning and explainable artificial intelligence can accelerate the pace of medical discoveries and ensure that such advancements are in accordance with the ethical needs of both practitioners and patients.

In sum, this review highlights the ethical importance of explainability when deploying AI systems in healthcare. Precision medicine and patient-centric approaches to healthcare that are driven by AI must be transparent to be trusted. In the future, AI and human expertise will be working in tandem to deliver personalized and ethical healthcare solutions. The implementation of AI systems by physicians is limited by the transparency of the systems and their ability to be understood [68]. However, explainable artificial intelligence can help forge the path towards building trust in precision medicine based on digital health data [84]. The widespread adoption of machine-learning models using digital health data for precision medicine is hindered by the slow progress in developing explainable methods [85]. Thus, integrating explainable artificial-intelligence approaches into healthcare systems is one key to realizing the full potential of AI in precision medicine.

### 4.1. Limitations

The keywords used to find journal articles limited the topics to interpretable machine learning and/or explainable artificial intelligence. The discovered topics will not necessarily reflect all possible themes in the burgeoning field of artificial intelligence more broadly. The interested reader can see reviews with a broader focus on artificial intelligence and digital health or precision medicine [86,87]. Moreover, five articles in the initial search were published in journals behind a paywall, and not accessible despite contacting authors. The Supplementary Materials include a list of the articles not available, as well as the code for text processing and topic modeling.

As this systematic review was not aimed at quantifying the evidence for a specific effect, traditional risk assessment of bias in individual studies did not directly apply to our topic-modeling synthesis of text from journal articles for a systematic review. The goal of our systematic review was to identify patterns and themes across a body of literature rather than evaluate the methodological quality of individual studies. Nonetheless, our study meets benchmark questions used to assess the overall quality of systematic reviews [88]. There were clear inclusion and exclusion criteria relevant for tapping the appropriate scientific literature, and a comprehensive literature search, unrestricted by time; our topic modeling of journal articles ensured all selected papers were adequately encoded.

### 4.2. Future Directions

Researchers at the crossroads of digital health and precision medicine should strive to understand their artificial-intelligence applications. For example, explainable artificial-intelligence approaches could help advance biophysical models and understanding of biological processes, as well as improve trust in using artificial-intelligence applications with digital health data to make medical decisions [82]. A barrier to progress is that machine-learning models need big data, yet repositories of publicly available digital health data are limited. Future studies using artificial intelligence should collect a multi-site, nationally representative sample that provides publicly available data from different digital health domains [89]. Ultimately, these endeavors could result in a transparent artificial-intelligence system utilizing digital health data for precision medicine.

The future of personalized medicine appears to be increasing the trustworthiness of AI systems by making them explainable. There are policy implications for how explainable methods can help meet regulations and policies regarding transparency. Future research could include multi-site studies that validate local explaining methods that make reliable predictions at the patient level. The end product of these studies should include some applications for healthcare workers that visualize explanations for diagnostic or treatment planning. Such multi-site studies could also help encourage collaboration across different areas of expertise as the use of artificial intelligence in healthcare grows.

## 5. Conclusions

This paper provides an up-to-date assessment of themes in research related to explainable artificial intelligence, digital health, and precision medicine. The potential contributions of explainable artificial intelligence to precision medicine span both theoretical and translational aspects. For example, explainable artificial intelligence holds promise for both enhancing our comprehension of disease mechanisms and visualizing regions of medical images important for making a diagnosis. In general, the convergence with digital health is in its early stages, yet precision medicine stands to benefit in many ways by embracing explainable artificial intelligence.

## Figures and Tables

**Table 1:** List of selected journal articles.

Author	Year	Title	Publication Title
Evans et al.	2018	The Challenge of Regulating Clinical Decision Support Software After 21st Century Cures	American Journal of Law & Medicine
Adadi et al.	2019	Gastroenterology Meets Machine Learning: Status Quo and Quo Vadis	Advances in bioinformatics
Shin et al.	2019	Current Status and Future Direction of Digital Health in Korea	The Korean Journal of Physiology& Pharmacology
Ahirwar et al.	2020	Interpretable Machine Learning in Health Care: Survey and Discussions	International Journal of Innovative Research in Technology and Management
Coppola et al.	2021	Human, All Too Human? An All-Around Appraisal of The “Artificial Intelligence Revolution” in Medical Imaging	Frontiers in Psychology
Wickramasinghe et al.	2021	A Vision for Leveraging the Concept of Digital Twins to Support the Provision of Personalized Cancer Care	IEEE Internet Computing
Bhatt et al.	2022	Emerging Artificial Intelligence–Empowered mHealth: Scoping Review	JMIR mHealth and uHealth
Chun et al.	2022	Prediction of Conversion to Dementia Using Interpretable Machine Learning in Patients with Amnestic Mild Cognitive Impairment	Frontiers in Aging Neuroscience
Gerussi et al.	2022	Artificial Intelligence for Precision Medicine in Autoimmune Liver Disease	Frontiers in Immunology
Iqbal et al.	2022	The Use and Ethics of Digital Twins in Medicine	Journal of Law, Medicine & Ethics
Ishengoma et al.	2022	Artificial Intelligence in Digital Health: Issues and Dimensions of Ethical Concerns	Innovación y Software
Khanna et al.	2022	Economics of Artificial Intelligence in Healthcare: Diagnosis vs. Treatment	Healthcare
Kline et al.	2022	Multimodal Machine Learning in Precision Health: A Scoping Review	npj Digital Medicine
Laccourreye et al.	2022	Explainable Machine Learning for Longitudinal Multi-Omic Microbiome	Mathematics
Roy et al.	2022	Demystifying Supervised Learning in Healthcare 4.0: A New Reality of Transforming Diagnostic Medicine	Diagnostics
Shazly et al.	2022	Introduction to Machine Learning in Obstetrics and Gynecology	Obstetrics & Gynecology
Wellnhofer et al.	2022	Real-World and Regulatory Perspectives of Artificial Intelligence in Cardiovascular Imaging	Frontiers in Cardiovascular Medicine
Wesołowski et al.	2022	An Explainable Artificial Intelligence Approach for Predicting Cardiovascular Outcomes Using Electronic Health Records	PLOS digital health
Albahri et al.	2023	A Systematic Review of Trustworthy and Explainable Artificial Intelligence in Healthcare: Assessment of Quality, Bias Risk, and Data Fusion	Information Fusion
Baumgartner et al.	2023	Fair and Equitable AI in Biomedical Research and Healthcare: Social Science Perspectives	Artificial Intelligence in Medicine
Bharati et al.	2023	A Review on Explainable Artificial Intelligence for Healthcare: Why, How, and When?	IEEE Transactions on Artificial Intelligence
Hong et al.	2023	Overcoming the Challenges in the Development and Implementation of Artificial Intelligence in Radiology: A Comprehensive Review of Solutions Beyond Supervised Learning	Korean Journal of Radiology
King et al.	2023	What Works Where and How for Uptake and Impact of Artificial Intelligence in Pathology: Review of Theories for a Realist Evaluation	Journal of Medical Internet Research
Kuwaiti et al.	2023	A Review of the Role of Artificial Intelligence in Healthcare	Journal of Personalized Medicine
Narayan et al.	2023	A Strategic Research Framework for Defeating Diabetes in India: A 21st-Century Agenda	Journal of the Indian Institute of Science
Vorisek et al.	2023	Artificial Intelligence Bias in Health Care: Web-Based Survey	Journal of Medical Internet Research
Zafar et al.	2023	Reviewing Methods of Deep Learning for Intelligent Healthcare Systems in Genomics and Biomedicine	Biomedical Signal Processing and Control

## Data Availability

The data used in this report came from published articles that are copyrighted. However, the articles used are listed in the report.

## Supplementary Materials

The following supporting information can be downloaded at: https://osf.io/tpxh6. Code can be downloaded at https://zenodo.org/records/10398384.
